# Liquiritigenin Decreases Selective Molecular and Behavioral Effects of Cocaine in Rodents

**DOI:** 10.2174/157015911795017371

**Published:** 2011-03

**Authors:** E. Y Jang, M Hwang, S. S Yoon, J. R Lee, K. J Kim, H.-C Kim, C. H Yang

**Affiliations:** 1 Department of Physiology, College of Oriental Medicine, Daegu Haany University, Daegu 706-828, South Korea; 2 The Research Center for Biomedical Resource of Oriental Medicine, Daegu Haany University, Daegu 706-828, South Korea; 3 Neuropsychopharmacology and Toxicology Program, College of Pharmacy, Kangwon National University, Chunchon 200-701, South Korea

**Keywords:** Liquiritigenin, cocaine, hyperlocomotion, CREB, c-Fos, nucleus accumbens, striatum.

## Abstract

Cocaine, as an indirect dopamine agonist, induces selective behavioral and physiological events such as hyperlocomotion and dopamine release. These changes are considered as consequences of cocaine-induced molecular adaptation such as CREB and c-Fos. Recently, methanolic extracts from licorice was reported to decrease cocaine-induced dopamine release and c-Fos expression in the nucleus accumbens. In the present study, we investigated the effects of liquiritigenin (LQ), a main compound of licorice, on acute cocaine-induced behavioral and molecular changes in rats. LQ attenuated acute cocaine-induced hyperlocomotion in dose-dependent manner. In addition, LQ inhibited CREB phosphorylation and c-Fos expression in the striatum and the nucleus accumbens induced by acute cocaine. Results provide strong evidence that LQ effectively attenuates the acute behavioral effects of cocaine exposure and prevents the induction of selective neuroadaptive changes in dopaminergic signaling pathways. Further investigation of LQ from licorice extract might provide a novel therapeutic strategy for the treatment of cocaine addiction.

## INTRODUCTION

Cocaine has been known as an indirect dopamine agonist to inhibit dopamine reuptake. It causes the release of dopamine in synaptic area of the mesolimbic and nigrostriatal DA system as a major mediator in the behavioral and reinforcing effects of cocaine [1]. In rodents, acute cocaine administration results in consequences of behavioral changes such as increase of locomotor activity, whereas chronic cocaine induces locomotor sensitization [2]. In the meantime, a neurochemical study has demonstrated that cocaine leads to the upregulation of several molecular markers such as c-Fos through the activation of postsynaptic D1/D2-like dopamine receptor [3]. c-Fos is a transcription factor which is one of the immediate early markers produced by acute cocaine. It is rapidly and transiently up-regulated for postsynaptic activation in dopaminergic target area including the striatum and the nucleus accumbens following acute cocaine [4]. In cocaine-treated brain, the expression of c-Fos is modulated by the activation of cAMP response elements binding (CREB). CREB is a transcription factor which binds to certain DNA sequences called cAMP response elements (CRE) and modulates the transcription of certain genes in response to cellular stimuli. For example, acute cocaine administration induces the phosphorylation of CREB, which in turn leads to the expression of immediate early marker, c-Fos [5]. Moreover, results of some animal studies provide evidence for the behaviors of cocaine. Infusion of CREB antisense oligonucleotide into the nucleus accumebns attenuates cocaine self-administration [6]. Microinjection of c-Fos antisense into the nucleus accumbens prevented cocaine-induced locomotor activity [7]. Based on these observations, it is suggested that the behavioral stimulation induced by the cocaine challenge is associated with extracellular DA release or postsynaptic gene expression.

*Glycyrrhizae radix* (licorice) is one of the oldest and most frequently used herbal medicines. *Glycyrrhizae radix* comprises flavonoids and pentacyclic triterpene saponin as major constituents such as liquiritin, liquiritigenin (LQ), isoliquiritigenin (ISL), glycyrrhizin and glycyrrhizic acid [8]. LQ, an isoform of ISL, is one of the flavonoids in *Glycyrrhizae radix.* LQ has been shown to exert cytoprotective effects against heavy metal-induced toxicity in cultured cells [9] and neuroprotective effect against beta-amyloid peptide in rat hippocampal neurons [10]. More importantly, our previous study demonstrated that methanolic extract of *Glycyrrhizae radix* or ISL significantly decreased dopamine release in the nucleus accumbens, locomotor activity, and c-Fos gene expression induced by acute cocaine challenge [4, 11]. These results raise the possibility that LQ may play an important role in modulating behavioral and molecular changes induced by cocaine. Thus, the present study was designed to investigate the effects of LQ on acute cocaine-induced locomotor activity and gene expression in rat brain.

## MATERIALS AND METHODS

### Animals

Male Sprague-Dawley rats (Daehan Animal, Seoul, Korea) were used weighing 260-300 g at the start of the experiment. All rats were kept on *ad libitum* food and water, and maintained under standard conditions (room temperature: 21 ± 2°C, relative humidity: 55-65%, light-dark cycle: 12 h). All procedures were carried out in compliance with the animal care guide-lines of the National Institutes of Health (NIH) “Guide for the Care and Use of Laboratory Animals.”

### Preparation of Liquiritigenin

LQ was obtained as described previously [9] and dissolved in vehicle. Vehicle is composed of polyethylene glycol/10% Tween 80/ethanol/ saline (7:1:1:1, v/v).

### Locomotor Activity

Locomotor activity was assessed in a rectangular container (40×40×45 cm) using a video-tracking system linked to the Ethovision program (Noldus Information Technology BV, Wageningen, Netherlands). The walls and floor were made of a clear plexiglas and painted black. Animals were allowed a 2-h habituation period in the activity containers and then orally given vehicle or LQ at doses of 1.25 (*n* = 5), 2.5 (*n* = 7), 5.0 (*n* = 9) mg/kg. After 1-h recording period, rats were treated with 20 mg/kg of cocaine hydrochloride (Macharlan, Smiss Limit, UK, *n* = 9) or saline (*n* = 6) by intraperitoneal injection, and the distance they traveled was measured for another 1-h period. In order to investigate the effect of LQ alone on the locomotor activity, rats were given LQ (5.0 mg/kg, p.o., *n* = 4) prior to saline administration. The locomotor activity was measured between 9 a.m. and 5 p.m.

### Immunohistochemistry

CREB phosphorylation and c-Fos expression were measured using different groups of rats (*n* = 3 per group) using the same cocaine challenge design. After cocaine (20 mg/kg, i.p.) treatment, rats were anesthetized with sodium pentobarbital [80 mg/kg, i.p.; 2 h time point for c-Fos detection and 15 min time point for phosphorylated form of CREB (pCREB) detection]. Preparation of brain tissue for immunohistochemical study began with transcardial perfusion of ice-cold 4% paraformaldehyde solution in 0.1 M PBS (pH 7.4). The experimental procedures for detection of c-Fos and CREB were carried as described in our previous study (4). Sections were incubated with rabbit anti-c-Fos antibodies (1:1000; Santa Cruz Biotechnology, Santa Cruz, CA, USA) and pCREB antibodies (1:1000; Cell Signaling Technology, CA, USA) for 20 h at 4°C.

### Statistical Analysis

Statistical analysis of data was carried out using the SPSS 11.0 software programs. The behavioral and molecular data were statistically analyzed with one-way ANOVA and post-hoc Tukey tests to compare the experimental and control groups.

## RESULTS

**Fig. (1):**
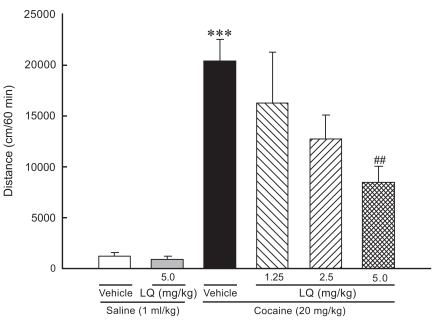
Effect of liquiritigenin (LQ) on locomotor activity of male Sprague-Dawley rats challenged with acute cocaine (20 mg/kg, i.p.) following oral administration of vehicle or LQ (1.25, 2.5, 5.0 mg/kg). Rats were also given LQ (5.0 mg/kg, p.o.) prior to saline administration. Results are expressed as mean ± SEM of total distance (cm) for 1 h immediately after cocaine or saline injection. Significant difference indicates as ^###^ P < 0.001 compared with saline and ** P < 0.01 compared with cocaine by post hoc Tukey’s test.

We evaluated the effect of LQ on locomotor activity in acute cocaine-treated rats. As shown in Fig. (**1**), challenge with cocaine (20395.0 ± 2110.4 cm) significantly produced a much larger increase in locomotor activity, compared to saline (1170.9 ± 351.8 cm). LQ at doses of 1.25, 2.5, 5 mg/kg decreased the locomotor activity induced by a systemic cocaine challenge to 16254.1 ± 4986.8 cm, 12718.1 ± 2365.5 cm, 8418.7 ± 1656.4 cm, respectively, indicating that LQ suppressed cocaine-induced hyperactivity in a dose-dependent fashion. LQ at a dose of 5 mg/kg significantly reduced the amount of cocaine-induced hyperactivity in 1-h session. To control for the possibility that LQ affects generalized suppression of locomotor activity, the treatment of LQ was carried out in saline-treated rats. Results showed that LQ at a dose of 5 mg/kg did not alter the locomotor activity in saline-treated rats (Fig. **1**).

**Fig. (2):**
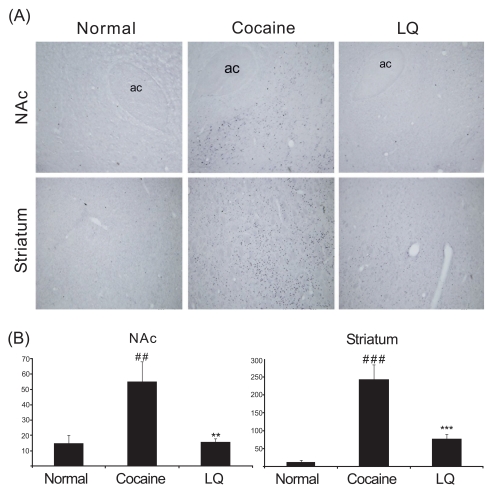
Effect of LQ on c-Fos expression in the nucleus accumbens (NAc) and striatum of male Sprague-Dawley rats challenged with acute cocaine (20 mg/kg, i.p.) following oral administration of vehicle or LQ (5.0 mg/kg). Representative photomicrographs (**A**) and quantitative analysis (**B**) of c-Fos-immunoreactive nuclei in the NAc and striatum (X 100). Significant differences indicate as ^###^ P < 0.001, ^##^ P < 0.01 compared with saline and *** P < 0.001, ** P < 0.01 compared with cocaine by post hoc Tukey’s test. ac: anterior commissure.

**Fig. (3):**
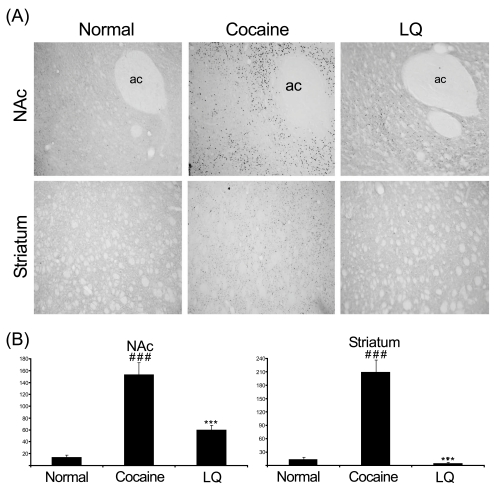
Effect of LQ on phospho-CREB expression in the nucleus accumbens (NAc) and striatum of male Sprague-Dawley rats challenged with acute cocaine (20 mg/kg, i.p.) following oral administration of vehicle or LQ (5.0 mg/kg). Representative photomicrographs (**A**) and quantitative analysis (**B**) of phospho-CREB immunoreactive signals in the NAc and striatum (X 100). Significant difference indicates as ### P < 0.001 compared with saline and *** P < 0.001 compared with cocaine by post hoc Tukey’s test. ac: anterior commissure.

To identify the involvement of molecular change in the mesolimbic dopamine system in the inhibition of hyperlocomotion by LQ, we examined the effect of LQ on the alteration of c-Fos expression and CREB activation in the striatum and the nucleus accumbens induced by acute cocaine in separate groups of rats. Similar to locomotor activity data, acute cocaine injection produced greater increases in phosphorylation of CREB and c-Fos expression than saline injection. In contrast, total CREB expression did not vary significantly by treatments (data not shown). Importantly, LQ at a dose of 5 mg/kg significantly decreased the phosphorylation of CREB and c-Fos expression induced by the systemic cocaine challenge (Figs. **2**, **3**).

## DISCUSSION

Many believe that cocaine acts to produce a large increase of extracellular dopamine in the mesolimbic dopamine pathway [12]. These cocaine-induced changes in brain levels of dopamine have long been associated with addictive behavior including locomotor activity, stereotype behavior and drug seeking- and taking-behavior [13, 14]. Accumulating evidence has established that a single cocaine administration induces rapidly and transiently expresses c-Fos *via* CREB activation at the postsynaptic dopaminergic area [5, 15, 16]. These molecular markers are synergistically enhanced by activating postsynaptic D1 and D2 dopamine receptors, the major projection area of the central dopamine system, however, it has been reported that D1, but not D2, receptor activation is sufficient for c-Fos expression [17-19]. The D1 dopamine receptor is widely expressed in the brain region and involved in mediating the rewarding properties of several drugs of abuse [20]. The activation of D1 dopamine receptor induces cyclic AMP *via* adenyl cyclase activation and protein kinase A and CREB phosphorylation at post-synapses. Phosphorylated CREB is translocated to the nucleus and then induces the expression of c-Fos. These effects have been confirmed in experimental studies showing that a selective D1 antagonist, SCH 23390 blocked the enhancement of pCREB and c-Fos expression in the striatum by acute cocaine [17] and CREB antisense prevented cocaine-induced c-Fos expression in dopamine intact striatum, but not in the dopamine-denervated striatum [21]. Accordingly, it is suggested that CREB may play a major role in the dopaminergic activation in cocaine-treated brain.

In agreement with these findings, our results showed that systemic challenge with cocaine hydrochloride produced a much larger increase in locomotor activity, c-Fos gene expression, and CREB phosphorylation in the striatum or the nucleus accumbens compared to systemic challenge with saline. Most importantly, results of the present study showed that LQ significantly attenuated the hyperactivity, c-Fos gene expression, and CREB phosphorylation in the striatum or the nucleus accumbens caused by acute cocaine injection. It has been demonstrated that cocaine-induced hyperactivity is closely associated with postsynaptic gene expression. For example, nucleus accumbens infusion of c-Fos antisense inhibited cocaine-induced locomotor stimulation [7]. Reinstatement of cocaine-seeking behavior by response-contingent cues enhanced c-Fos mRNA and protein expression in the striatum and the nucleus accumbens [22]. Local infusion of CREB antisense into the nucleus accumbens produced decrease in cocaine self-administration behavior [6]. A similar conclusion was obtained in another study in which locomotor activity and striatal CREB levels were decreased in mice lacking dopamine D1 receptor [15]. Thus, based on these studies, our results suggest the possibility that LQ prevented the expected cocaine-induced increase in locomotor activity through reduction of postsynaptic neuronal activity in the brain.

In conclusion, results provided strong evidence that LQ effectively reduced increases in locomotor activity, expression of c-Fos, and CREB phosphorylation in the striatum and the nucleus accumbens induced by a systemic cocaine challenge. These results suggest that LQ may be effective in inhibiting the behavioral effects of cocaine by modulating postsynaptic gene expression in the central dopaminergic system.
